# Production of Soluble Human Vascular Endothelial Growth Factor VEGF-A_165_-Heparin Binding Domain in *Escherichia coli*

**DOI:** 10.1371/journal.pone.0055690

**Published:** 2013-02-08

**Authors:** Arefeh Seyedarabi, Lili Cheng, Ian Zachary, Snezana Djordjevic

**Affiliations:** 1 Institute of Structural and Molecular Biology, University College London, London, United Kingdom; 2 Ark Therapeutics Ltd., London, United Kingdom; 3 Centre for Cardiovascular Biology and Medicine, British Heart Foundation Laboratories at University College London, London, United Kingdom; University of Nebraska Medical Center, United States of America

## Abstract

We report a method for production of soluble heparin binding domain (HBD) of human vascular endothelial growth factor VEGF-A_165_. Recombinant VEGF-A_165_-HBD that contains four disulphide bridges was expressed in specialised *E. coli* SHuffle cells and its activity has been confirmed through interactions with neuropilin and heparin. The ability to produce significant quantities of a soluble active form of VEGF-A_165_-HBD will enable further studies addressing the role of VEGF-A in essential processes such as angiogenesis, vasculogenesis and vascular permeability.

## Introduction

Vascular endothelial growth factor-A (VEGF-A), plays essential roles in vascular development in embryogenesis, in the maintenance of the normal function of the adult vasculature and in neovascularisation associated with cancer, eye and other diseases [1]. VEGF-A elicits cellular responses through binding to the receptor tyrosine kinases, VEGFR1 and VEGFR2 [1]. In addition, VEGF-A interacts with neuropilins (NRP) 1 and 2, transmembrane proteins that function as co-receptors to VEGFRs [2], [3].

VEGF-A is expressed in multiple isoforms (i.e. VEGF-A_121_, VEGF-A_145_, VEGF-A_165_, VEGF_165b_, VEGF-A_189_, and VEGF-A_206_) that are generated through alternative mRNA splicing of a single VEGF-A gene transcript containing 8 exons [4], [5], [6]. VEGF-A_165_ is the most abundant and biologically active isoform that is able to bind VEGFR1, VEGFR2, NRP1 and NRP2. VEGF-A_165_ also binds heparin and heparan sulfate, a property shared by VEGF-A_189_ and VEGF-A_206_ isoforms which are not diffusible and are thought to remain tightly associated with the cell surface or extracellular matrix [7]. All of these isoforms contain the 50 residues-long C-terminal protein region encoded by exons 7 and 8 that was identified as a ‘heparin-binding domain’ (HBD) [7]. In contrast, VEGF-A_121_, an isoform that does not include exon 7, does not bind to heparin and is freely diffusible. VEGF-A is a dimeric molecule, with each polypeptide chain containing multiple intrachain disulphide bonds forming a cysteine knot motif. Although, several structures of VEGF interacting with VEGFR extracellular Ig-like domains 2 and 3 have been reported, including the structure of a VEGF-A/VEGFR2 complex [8], none of these contains the heparin binding domain of VEGF. The structure of the C-terminal 55 residues of VEGF-A_165_ has been determined by solution NMR spectroscopy [9]. In that study the protein fragment that was generated by plasmin digestion of the refolded full-length VEGF-A_165_ includes additional 5 amino acids preceding HBD. The domain is very basic, with pI of ∼11, shares no sequence or structure homology to other known proteins and comprises two β-sheet subdomains, each containing two disulphide bridges. Molecular dynamics simulation and the NMR studies examining dynamic properties of the isolated HBD showed that the N-terminal region exhibited greater flexibility compared to the C-terminal subdomain [10], [11].

While heparin binding domain does not appear to bind to the soluble VEGF receptors [7], HBD binds to NRP and is required for VEGF-A_165_ interaction with NRP, either independently or when in a complex with NRP and VEGF receptors where VEGF-A_165_ bridges between VEGFR2 and NRP. It was shown that the C-terminal region of VEGF-A_165_ is critical for its mitogenic potency and it was postulated that its removal by plasmin activity might result in a weakening of the angiogenic signal further away from the site of VEGF synthesis [7]. However, very little is known about the activity of the plasmin released HBD. In NRP, the region comprising tandem coagulation factor V/VIII type domains b1 and b2 specifically interacts with the C-terminus of VEGF-A_165_ with the b1 domain of NRP playing the most important role in VEGF-A binding [12], [13]. In addition to interacting with VEGFs, NRPs bind to the class 3 semaphorin family of axon guidance molecules (SEMAs), unrelated to VEGFs. By signalling in response to these two families of ligands, NRPs play essential roles in embryonic blood vessel development and neuronal patterning. NRPs are also implicated in the pathogenesis of cancer and other diseases [14], [15].

A full understanding of the signaling properties, specificity and molecular basis of VEGF/NRP/VEGFR2 interactions has been hampered by an inability to easily produce a soluble VEGF-A HBD domain. To date, the production of soluble VEGF-A_165_ in *E. coli* involved denaturation, refolding and extensive purification from the insoluble protein fraction [9]. Refolded full-length VEGF-A_165_ has subsequently been used to generate the HBD through plasmin proteolytic cleavage [7], [9], [16]. A smaller C-terminal domain (CTD) region of VEGF-A_165,_ has been previously chemically synthesized [10], [17]. The NRP1 b1 domain has also been co-crystallised with a small molecule antagonist of the VEGF interaction with NRP1 [17]. Most recently, an attempt to gain an insight into the NRP/VEGF interaction was made by generating a fusion of the NRP1 b1 and VEGF-A_165_-HBD domains and determining the crystal structure of this chimerical protein construct [18]. While the structure of this fusion protein might provide some additional information, caution should be exercised in interpretation of specific points of interaction inferred from this structure as these are restricted by the artificial covalent attachment between the two domains.

Here we report a new method that overcomes the need to use refolding of an insoluble VEGF-A_165_ protein to obtain soluble VEGF-A_165_-HBD. The availability of soluble biologically active VEGF-A HBD will be invaluable for future structural elucidation of the interaction between VEGF-A and NRPs and will enable further studies addressing physiological role of HBD.

## Materials and Methods

### Expression and Purification of VEGF-A_165_-HBD

The gene sequence corresponding to the 50 residue-long HBD of VEGF-A_165_ encoded by exons 7 and 8 was adapted to the genome of E. coli K12 and synthesised by Eurofins (for details see below). The restriction enzyme sequences of NdeI (CATATG) and XhoI (CTCGAG) were incorporated into the 5′ and 3′ sites of the gene, respectively. The synthesized gene was then sub-cloned into pET14b (Novagen). The recombinant pET14b: *VEGF-A165-HBD* expression construct was confirmed by DNA sequencing (Eurofins) and transformed into Rosetta-gami 2 (DE3) pLysS cells (EMD Chemicals, #71403-3) and SHuffle cells (New England Biolabs, #C3029H). Tetracycline was used when growing Rosetta-gami 2 (DE3) pLysS cells in order to select only for mutations in thioredoxin reductase (*trxB*) and glutathione reductase (*gor*) genes that enhance disulphide bond formation in the cytoplasm [19]. Since only the features of the Origami 2 strain was selected from the Rosetta-gami 2 (DE3) pLysS cells, the cells used in producing VEGF-A_165_-HBD are referred to as Origami 2 cells. SHuffle cells were used as they are *E. coli* B cells engineered to form disulphide bonded proteins in the cytoplasm through the expression of a chromosomal copy of the disulphide bond isomerase DsbC that promotes the correction of mis-oxidized proteins into their correct form [19], [20]. In both cases, 10 ml overnight cultures were transferred into 1 liter lysogeny broth media (LB) and cells were grown at 37°C until absorbance at 600 nm reached 0.6. The cells were then induced with isopropyl-β-D-thio-galactoside (IPTG) at a final concentration of 0.5 mM and left overnight at 30°C. Cells were then centrifuged at 4,000 rpm for 15 minutes in a Beckman Coulter Avanti J-26 XP centrifuge (using a JLA-8.1000 rotor) and resuspended in buffer A consisting of 50 mM Tris pH 7.9, 30 mM imidazole and 300 mM NaCl. The cells were frozen at −20°C and subsequently thawed and sonicated. The soluble protein was separated from the insoluble fraction through centrifugation at 18,000 rpm for 30 minutes in a Sorvall RC 5B Plus centrifuge (using a SS-34 rotor). The soluble VEGF-A_165_-HBD was then purified via its hexahistidine tag (His_6_-tag) on a nickel chelating affinity column (GE Healthcare, #17-5248-02) attached to an AKTA prime FPLC instrument (GE Healthcare) using a gradient of 0–100% (v/v) buffer B consisting of 50 mM Tris pH 7.9, 600 mM imidazole and 300 mM NaCl. The fractions containing purified VEGF-A_165_-HBD were ultimately confirmed by SDS-PAGE analysis as the weak absorbance of VEGF-A_165_-HBD at 280 nm was masked by increasing amounts of imidazole. Biorad protein assay solution (Biorad, #500-0006) was initially used to decide which fractions contained eluted protein (1 ml of Biorad solution was diluted in a total volume of 5 ml and then aliquots of 50 µl were mixed with 20 µl of protein sample). A quick visible change of colour from brown to blue was used to detect presence of protein in the fractions using Biorad solution. VEGF-A_165_-HBD was further purified on a preparative HiLoad 16/60 Superdex 75 size exclusion column (GE Healthcare, #17-5174-01). The protein yield from a litre of culture was estimated to be 5 mg (1 mg of VEGF-A_165_-HBD from every g of SHuffle cell pellet). His_6_-tag of VEGF-A_165_-HBD was removed with thrombin (Novagen, #69671) in a cleavage buffer consisting of 20 mM Tris pH 8.4, 150 mM NaCl and 2.5 mM CaCl_2_. To every mg of protein, 1 µl of thrombin (1 unit/µl) was added and the mixture was dialysed overnight at room temperature using a Float-A-Lyzer G2 dialysis tube with MWCO of 0.5–1 kDa (Spectra/Por, #G235051). Thrombin was removed either by filtration or by passing the protein mixture over the heparin column. Purified protein is stored at −20°C.

### Gene Encoding *VEGF-A_165_ -HBD*

*Original sequence:*

CCC TGT GGG CCT TGC TCA GAG CGG AGA AAG CAT TTG TTT GTA CAA GAT CCG CAG ACG TGT AAA TGT TCC TGC AAA AAC ACA GAC TCG CGT TGC AAG GCG AGG CAG CTT GAG TTA AAC GAA CGT ACT TGC AGA TGT GAC AAG CCG AGG CGG TGA.

*Adapted sequence and restriction enzyme sites (in bold):*

**5′ CATATG** CCG TGT GGC CCA TGT TCG GAA CGT CGC AAA CAT CTG TTT GTG CAG GAT CCG CAA ACG TGC AAA TGC AGC TGC AAG AAC ACC GAT AGT CGC TGC AAA GCG CGT CAG TTA GAG CTG AAT GAA CGG ACT TGT CGC TGT GAC AAA CCT CGT CGC TAA **CTCGAG** **3′**.

### Binding of VEGF-A_165_-HBD to Heparin Column

VEGF-A_165_-HBD was shown to bind to heparin using a heparin column (GE Healthcare- 5 ml column, 17-0407-01) and eluted at a concentration of 500 mM NaCl. The Heparin column had covalently bound heparin, a naturally occurring sulphated glucosaminoglycan that is extracted from the native proteoglycan of porcine intestinal mucosa, with alternating units of uronic acid and D-glucosamine, most of which are substituted with one or two sulphate groups.

### Binding of VEGF-A_165_-HBD to NRP in a Cell Based Assay

Binding displacement experiments were carried out using human prostate cancer cell line DU145 as previously described [17], [21]. 96-well plates were pre-coated with PDL (Poly-D-Lysine) for one hour and washed three times with distilled water. DU145 cells were plated at 2×10^4^ per well in 0.1 ml medium and after four hours, were infected with NRP1 adenovirus (Ad.NRP1). The binding assay was performed 48 hours after adenoviral infection by the addition of various concentrations of either VEGF-A_165_ (R&D Systems) (positive control) or VEGF-A_165_-HBD diluted in binding medium with or without 0.1% bovine serum albumin (BSA), followed by addition of 1 nM biotinylated (bt) VEGF-A_165_ (R&D Systems). DU145 cells used in these assays do not express other VEGF receptors [21] and the levels of endogenously expressed NRP1 are too low to reliably measure binding of bt-VEGF-A_165_ to endogenous NRP1. After 2 h of incubation at room temperature, the plates were washed three times with phosphate buffered saline (PBS). The bt-VEGF-A_165_ bound to Ad.NRP1 was detected by incubation with streptavidin-horseradish peroxidase conjugates for 30 min. Cells were then washed 3 times in PBS before detection with substrate reagent (R&D Systems) for 20 min. The reaction was stopped with stopping solutions and signal intensity quantified using a Tecan Genios plate reader at A450 nm with a reference wavelength of 595 nm. Nonspecific binding was determined in the presence of 100-fold excess of unlabelled VEGF-A_165_.

### Binding Assay of VEGF-A_165_-HBD to NRP Using Size Exclusion Chromatography

VEGF-AVEGF-A_165_-HBD binds to NRP in solution as demonstrated by both a preparative HiLoad 16/60 Superdex 75 size exclusion chromatography column (GE Healthcare, #17-5174-01) and an analytical Superdex 75 pc 3.2/30 size exclusion column (GE Healthcare, #17-0771-01). This observation is also not dependent on presence of the N-terminal affinity tag. Binding of NRP1 and NRP2 b1 domains to VEGF-A_165_-HBD was assessed by mixing a tenfold excess of VEGF-A_165_-HBD with NRP1 and NRP2 b1 domains (at concentrations of 0.6 mM VEGF-A_165_-HBD and 0.06 mM NRP b1). All proteins were purified and dialysed in buffer consisting of 50 mM Tris pH 7.9 and 100 mM NaCl prior to mixing. The protein mixtures were incubated at room temperature for 30 minutes and then loaded onto a preparative Superdex 75 column. The eluted fractions from a single peak were analysed by SDS-PAGE which revealed the presence of both VEGF-A_165_-HBD and NRP1 or NRP2 b1 domains. Further analysis of VEGF-A_165_-HBD/NRP interaction was carried out by using analytical size exclusion chromatography. VEGF-A_165_-HBD, NRP b1 and b1b2 domains were purified and dialysed in buffer consisting of 50 mM Tris pH 7.9 and 100 mM NaCl. To test the binding of VEGF-A_165_-HBD to NRP b1 domain, a molar ratio of 1∶10 (NRP: VEGF-A_165_-HBD) was used and the mixture incubated at room temperature for an hour. Based on the elution profiles from the analytical Superdex 75 column it was apparent that the eluting peak for the mixture had shifted to the left (in comparison to the NRP b1 peak) indicating that the complex had formed. A similar approach was taken to test the binding of VEGF-A_165_-HBD and NRP b1b2 domain except that the molar ratio used was 1∶28.

### Over-expression and Purification of NRP1 and NRP2 Domains Used in this Study

Recombinant pET15b-TEV:*nrp1-b1*, pET15b-TEV:*nrp2-b1* and pET15b-TEV:*nrp1-b1b2* constructs were made and gene sequences confirmed by DNA sequencing (Eurofins). The constructs were then transformed into Rosetta-gami 2 (DE3) pLysS cells (EMD, #71403-3). Tetracyclin and Chloramphenicol were used when growing Rosetta-gami 2 (DE3) pLysS cells in order to select for mutations in thioredoxin reductase (*trxB*) and glutathione reductase (*gor*) genes that enhance disulphide bond formation in the cytoplasm [19] as well as providing seven tRNAs accounting for the rare codon problem in *E. coli* [22], [23], [24], [25], [26]. To over-produce the proteins, 10 ml overnight cultures were transferred into 1 litre LB media and cells grown at 37°C until absorbance at 600 nm reached 0.6. The cells were then induced with IPTG at a final concentration of 0.5 mM and left overnight at 30°C. Cells were centrifuged at 4,000 rpm for 15 minutes in a Beckman Coulter Avanti J-26 XP centrifuge (using a JLA-8.1000 rotor) and then resuspended in buffer A consisting of 50 mM Tris pH 7.9, 30 mM imidazole and 300 mM NaCl. The cells were then frozen at −20°C and subsequently thawed and sonicated. The soluble protein was separated from the insoluble fraction through centrifugation at 18,000 rpm for 30 minutes in a Sorvall RC 5B Plus centrifuge (using a SS-34 rotor). The soluble proteins were purified via their His_6_-tag on a nickel chelating affinity column (GE Healthcare, #17-5248-02) attached to an AKTA prime FPLC instrument (GE Healthcare) using a gradient of 0–100% (v/v) buffer B consisting of 50 mM Tris pH 7.9, 400 mM imidazole and 300 mM NaCl. To remove the His_6_-tag from NRP1 b1, NRP2 b1 and NRP1 b1b2 proteins, fractions eluted from the nickel chelating column were pooled and the His_6_-tagged TEV protease added (1 mg of TEV was used per 8 mg of protein) in the presence of 0.3 mM *L*-cysteine and 3 mM *L*-cystine. The proteins were then dialysed overnight at 4°C in buffer containing 50 mM Tris pH 7.9, 20 mM imidazole, 300 mM NaCl, 0.3 mM *L*-cysteine and 3 mM *L*-cystine. The dialysed proteins were subsequently applied to nickel chelating beads in solution to separate His-cleaved proteins from His_6_-tagged TEV protease which bound onto the beads.

## Results and Discussion

**Figure 1:**
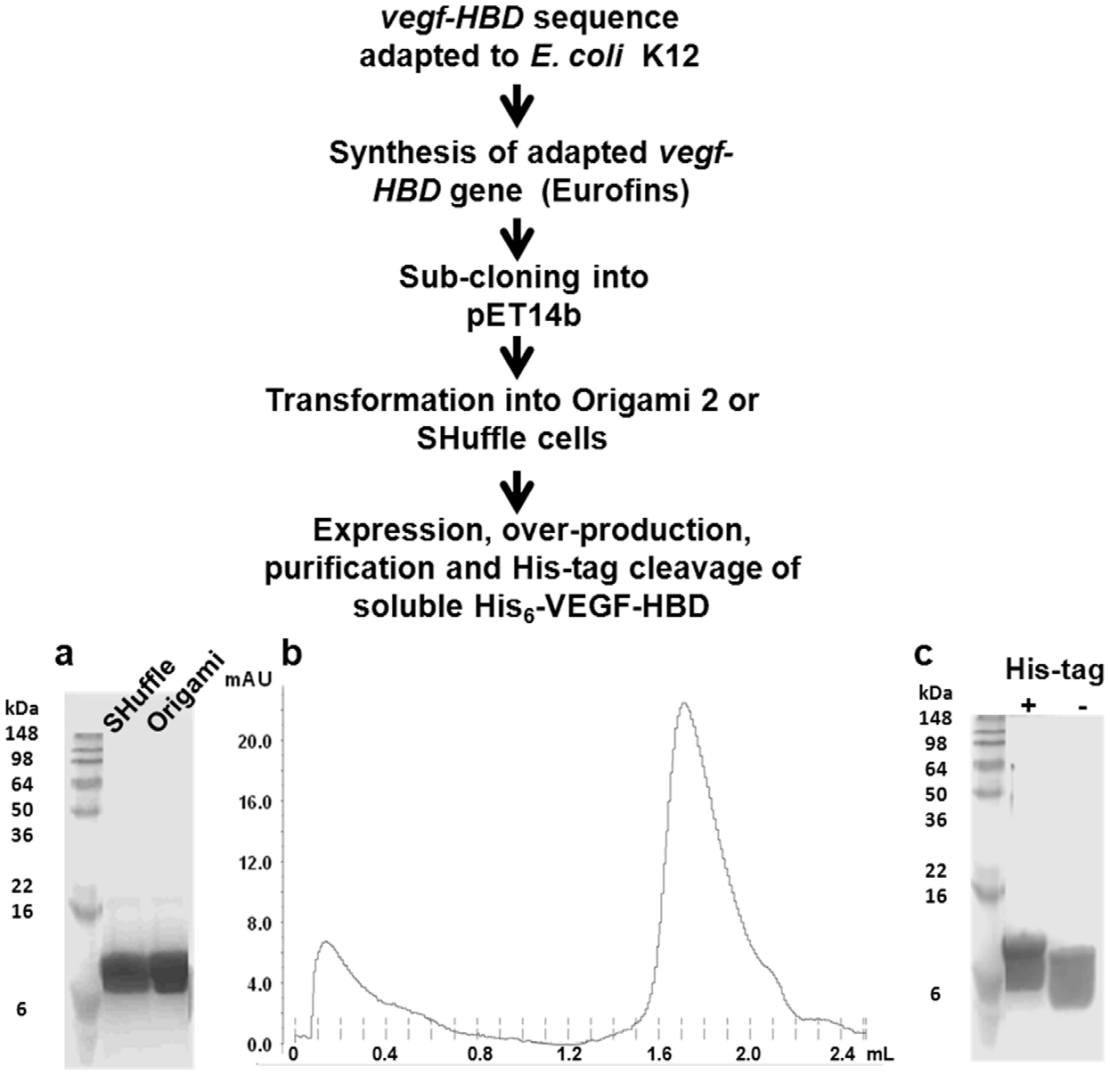
Steps to producing soluble VEGF-A_165_-HBD. The gene sequence encoding the VEGF-A_165_-HBD protein was adapted to that of *E. coli* K12 genome and the 5′ and 3′ restriction enzyme sequences of *Nde*I and *Xho*I were incorporated, respectively. The gene was then synthesised and subcloned into pET14b by Eurofins. The recombinant plasmid was transformed into Origami 2 and SHuffle cells and tested for expression. (a) VEGF-A_165_-HBD was over-produced and purified from both cell types; however, VEGF-A_165_-HBD produced from SHuffle cells showed a greater yield of presumably folded protein based on absorbance at 280 nm which was solely dependent on cysteines having formed cystines (disulphide bonds). Even though relative staining of the protein bands on the gel would suggest that the two protein samples are at similar concentrations, absorbance measurements at 280 nm indicate that the sample from SHuffle cells had a concentration of 3.1 mg/ml of disulphide-bonds-containing protein compared to 1.7 mg/ml in Origami cells. (b) VEGF-A_165_-HBD protein from SHuffle cells was further purified on a preparative Superdex 75 size exclusion column. (c) His_6_-tag was successfully removed using thrombin and resulting VEGF-A_165_-HBD retained solubility and stability as assessed by absence of precipitation and further proteolytic cleavage.

VEGF-A_165_-HBD was purified from the soluble cell extracts. BioRad protein assay reagent, based on the method of Bradford, as well as sodium dodecyl sulphate polyacrylamide gel electrophoresis (SDS-PAGE) analyses, was used to identify the fractions containing purified VEGF-A_165_-HBD. VEGF-A_165_-HBD contains no tryptophan or tyrosine residues whose property to absorb light at 280 nm is commonly used to determine concentration of proteins in solution. In the case of VEGF-A_165_-HBD only weak contribution to absorbance at 280 nm could come from cystines – cysteine residues oxidized to form disulphide bonds [27]. There are eight cysteine side chains in the expressed VEGF-A_165_-HBD protein and if we were to assume that all four disulphide bridges were formed in VEGF-A_165_-HBD, the calculated extinction coefficient [27] would be 500 M^−1^Lcm^−1^. While the formation of disulphide bonds is not sufficient evidence that a native-like and active conformation was generated, we took the appearance of the disulphide bonds, consistent with the formation of the three-dimensional structure as the initial measure of folded state of the produced protein. Thus, when the two protein samples, whose concentration was determined by a traditional Bradford protein assay to be equal, exhibited differences in A_280_ it was interpreted as if the protein sample with the higher A_280_ value had a higher disulphide bond content and higher proportion of folded protein. Following protein purification by metal ion affinity chromatography there was a noticeable difference in absorbance at 280 nm per unit of the protein concentration for a protein sample generated in SHuffle cells, compared to the A_280_ absorbance of an equivalent concentration (as judged by protein assay and the SDS-PAGE) of a protein produced in Origami 2 cells. Fig. 1a. shows Coomassie Blue stained SDS-PAGE gel of the protein samples purified from the two cell types. Although the two protein bands exhibit similar levels of staining, the observed absorbance at 280 nm for the two protein samples suggests that the sample from SHuffle cells with A_280_ of 0.2 contained a 3.2 mg/ml of folded protein compared with an estimated folded protein concentration of 1.6 mg/ml (A_280_ = 0.1) in protein sample purified from the Origami 2 cells. This observation indicated that SHuffle cells were more efficient in producing higher yields of folded and disulphide-linked VEGF-A_165_-HBD. It should be noted that protein bands on the SDS-PAGE gels occasionally had smeared appearance due to transient disulphide bonds formation during the electrophoresis.

Even though the presence of disulphide bonds might be associated with the folding of the polypeptide only the right pairing of the cysteine residues would give rise to a functionally active protein structure. Heparin binding of the recombinant VEGF-A_165_-HBD was confirmed through interaction with a heparin column; bound protein eluted at 500 mM NaCl. Interestingly, when VEGF-A_165_-HBD produced from Origami 2 cells was passed over a heparin column, the resulting eluted protein had a higher absorbance at 280 nm indicating that heparin binding facilitated correct disulphide bond formation in VEGF-A_165_-HBD. VEGF-A_165_-HBD protein produced from SHuffle cells was further purified by size exclusion chromatography (Fig. 1b) and subsequent removal of the His_6_-tag by thrombin. The resulting VEGF-A_165_-HBD remained both soluble and stable in solution (Fig. 1c). The protein showed no significant signs of degradation or precipitation after several weeks at 4°C, however we have routinely stored the purified protein at −20°C.

**Figure 2:**
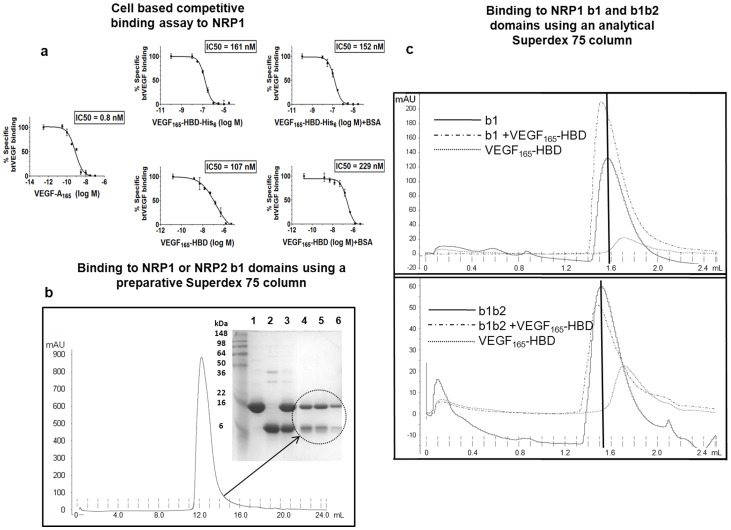
Binding of VEGF-A_165_-HBD to NRP as detected by a cell based assay and size exclusion chromatography. (a) Binding of VEGF-A_165_-HBD to NRP1 was determined in DU145 cells expressing an adenoviral construct encoding NRP1 [17]. These cells do not express other VEGF receptors [21]. Cells were incubated with biotinylated VEGF-A_165_ in the presence of the indicated concentrations of either unlabelled VEGF-A_165_ (positive control), or VEGF-A_165_-HBD containing a His_6_tag or VEGF-A_165_-HBD without the tag. Values presented are the means (±SEM) obtained from two independent experiments. Other experimental details are described in Materials and Methods. (b) Binding of VEGF-A_165_-HBD to b1 domains of NRP1 and NRP2 was assessed by size exclusion chromatography. The protein mixtures of a tenfold molar excess of VEGF-A_165_-HBD with the purified b1 domain from either NRP1 or NRP2 were incubated at room temperature for 30 minutes. The FPLC profile for VEGF-A_165_-HBD and NRP1 b1 mixture is shown. SDS-PAGE analysis was used to evaluate samples of NRP1 b1, VEGF-A_165_-HBD, and the protein mixture before being loaded onto the size exclusion column (lanes 1–3, respectively) as well as samples of fractions which eluted from the single peak (lanes 4–6), showing that VEGF-A_165_-HBD and NRP1 b1 co-eluted from the preparative Superdex 75 column. A similar result was seen for VEGF-A_165_-HBD and NRP2 b1. (c) Formation of the molecular complexes was also investigated by analytical size exclusion chromatography. NRP1 b1 and b1b2 domains as well VEGF-A_165_-HBD were initially loaded separately onto the analytical Superdex 75 column and their corresponding FPLC traces are shown in solid and dashed lines, respectively. To detect binding, NRP1 b1 or b1b2 domains were mixed with VEGF-A_165_-HBD in solution at a molar ratio of 1∶10 and 1∶28, respectively and incubated for an hour at room temperature. The mixtures were then applied to the column. The main peaks in the elution profiles revealed a shift to the left of the peak positions corresponding to the unbound NRP1 b1 or b1b2 domains (indicated by vertical black lines), suggesting complex formation.

Crucially, both His_6_-tagged VEGF-A_165_-HBD and VEGF-A_165_-HBD with the His_6_-tag removed bind NRP1 in a cell based binding assay (Fig. 2a) [17]. As it was previously shown that the C-terminal HBD domain of VEGF-A_165_ is required for its biological activity via interaction with NRPs [7] we tested the potential of the recombinant VEGF-A_165_-HBD to displace VEGF-A_165_ from the surface of DU145 cells. These cells do not express other VEGF receptors and the binding of VEGF-A_165_ was due to the presence of the adenovirully expressed NRP1 [17], [21]. The apparent binding affinity of VEGF-A_165_-HBD for NRP1, as judged by IC50s, was between 100–200 nM which is in agreement with the previously reported values [17], [28], [29] and it was unaffected by the presence of BSA in the binding buffer.

Further, we demonstrated binding of VEGF-A_165_-HBD to purified recombinant NRP1 b1 and NRP2 b1 domains through size exclusion chromatography column, as the samples eluted (from a single peak) contained both proteins (Fig. 2b). Additionally, experiments using an analytical size exclusion column confirmed the binding of NRP1 b1 and b1b2 domains to VEGF-A_165_-HBD (Fig. 2c). Molecular complex between NRP domains and VEGF-A_165_-HBD eluted earlier from the size exclusion column compared to the elution volumes for the protein peaks of NRP1 b1 and b1b2 domains as well as the elution peak for the free VEGF-A_165_-HBD (Fig. 2c). Interaction between NRP and VEGF-A_165_-HBD was also observed in affinity chromatography protocols where His_6_-tagged NRP1 b1 domain that was bound to the Ni-NTA column was used to capture un-tagged VEGF-A_165_-HBD from the solution; the two proteins co-eluted upon addition of the imidazole.

The approach taken in this study to produce soluble VEGF-A_165_-HBD can be used to further our understanding of the interaction between this domain, heparin and NRP receptors. In particular, it opens the path for carrying out structural, functional and thermodynamic analyses of complexes with VEGF-A_165_-HBD receptors. Characterizations of VEGF-A_165_-HBD interactions with its binding partners will ultimately be instrumental in the development of novel therapeutic molecules targeted to interfere with these interactions.
